# Protecting nonlocal quantum correlations in correlated squeezed generalized amplitude damping channel

**DOI:** 10.1038/s41598-022-24789-z

**Published:** 2022-11-28

**Authors:** Shuo Wang, Xin-Hong Han, Wei-Chen Li, Tian Qian, Xuan Fan, Ya Xiao, Yong-Jian Gu

**Affiliations:** College of Physics and Optoelectronic Engineering, Ocean University of China, Qingdao, 266100 People’s Republic of China

**Keywords:** Physics, Quantum physics, Quantum information

## Abstract

Nonlocal quantum correlations, such as quantum entanglement, quantum steering, and Bell nonlocality, are crucial resources for quantum information tasks. How to protect these quantum resources from decoherence is one of the most urgent problems to be solved. Here, we investigate the evolution of these correlations in the correlated squeezed generalized amplitude damping (SGAD) channel and propose a scheme to protect them with weak measurement (WM) and quantum measurement reversal (QMR). Compared with the results of the uncorrelated SGAD channel, we find that when $n=1$, correlation and squeezing effects can prolong the survival time of quantum entanglement, Bell nonlocality, and quantum steering by about 152 times, 207 times, and 10 times, respectively. In addition, local WM and QMR can effectively recover the disappeared nonlocal quantum correlations either in uncorrelated or completely correlated SGAD channels. Moreover, we find that these initial nonlocal quantum correlations could be drastically amplified under the correlated channel. And the steering direction can be flexibly manipulated either by changing the channel parameters or the strength of WM and QMR. These results not only make a step forward in suppressing decoherence and enhancing quantum correlation in noise channels, but also help to develop relevant practical applications.

## Introduction

Nonlocality, a distinctive feature that distinguishes the quantum world from the classical one, describes the ability of objects to perform “spooky action at a distance”. Quantum entanglement, quantum steering, and Bell nonlocality are three typical nonlocal quantum correlations that originate from the famous “EPR Paradox”^1^. In this paradox, Einstein, Podolski, and Rosen pointed out that there is a contradiction between local realism and the completeness of quantum mechanics. In response, Schrödinger introduced the concept of quantum steering^2,3^. To rule out the existence of the local hidden variable (LHV) model in the paradox, Bell provided an experimental criterion, called Bell inequality, in 1964^4^. And the corresponding nonlocal correlation is called Bell nonlocality. Later, Clauser, Horne, Shimony, and Holt refined the Bell inequality to a more experimental-friendly CHSH inequality^5^, opening an epoch of unrelenting exploration of nonlocal quantum correlations. In 1989, Werner found a class of inseparable mixed states that exhibits nonlocal effects even though it cannot violate Bell’s inequality, which is defined as quantum entanglement^6^. Subsequently, a series of experimental criteria for entanglement were proposed^7–10^. Bell nonlocality and quantum entanglement have made great progress both theoretically and experimentally between 1964 and 2006. However, quantum steering has not attracted wide attention until 2007, when Wiseman et al. redefined it strictly and presented detecting criterion^11^. They point out that quantum steering denotes a quantum correlation cannot be reproduced in terms of LHV-LHS model, where “LHS” stands for “local hidden state”^11^. It is different from Bell nonlocality, which does not admit a LHV-LHV model, and quantum entangled, which cannot be described by a LHS-LHS model. The relationship between the above three kinds of nonlocal quantum correlations is that quantum steering stands between Bell nonlocality and quantum entanglement, and exhibits particular asymmetry^12–16^. These nonlocal quantum correlations are very important resources which have a vast range of information in quantum information tasks: quantum entanglement can be applied to quantum communication, quantum computing and quantum metrology^17,18^, quantum steering can be further used to deal with one-sided device-independent tasks^19,20^, Bell nonlocality can even be used for full device-independent tasks, such as quantum teleportation^21^, quantum key distribution^22–25^ and quantum secure direct communication^26–33^.

However, these nonlocal quantum correlations are very fragile and easily degraded by the unavoidable interaction between the system and its surrounding environment^34^. And the system–environment interactions can be described as quantum channels^34^. One of the simplest example is the amplitude damping (AD) channel, which is a prototype model of a dissipating interaction between the quantum system and its zero-temperature environment^35^. In general, the real physical environment is the thermal bath, and the dissipative interaction is represented as a generalized amplitude damping (GAD) channel^35^. In addition, including the effect of squeezing, the GAD channel can be further expanded to a squeezed generalized amplitude damping (SGAD) channel^36^. What’s more, taking the correlation time of the environment and the time between the uses of successive channels into consideration, the SGAD channel can be further divided into correlated and uncorrelated^37–40^.

In recent years, the influence of noise channels on the evolution of nonlocal quantum correlation has been widely studied^41–43^. However, most of them are either limited to one of the nonlocal quantum correlations or ignored the correlation and squeezing effects of quantum channels. In addition, it is essential to recover the nonlocal quantum correlations when destruction happens. Numerous methods have been proposed to improve the nonlocal quantum correlations quality, such as entanglement purification^44–46^, weak measurement(WM) and quantum measurement reversal (QMR)^47–51^. Especially, WM and QMR has been demonstrated to effectively overcome the degenerative influence of AD channel^47–51^. However, whether the destroyed nonlocal quantum correlations in the correlated SGAD channel can be recovered via local WM and QMR remains unknown.

In this paper, we investigate the effects of correlated SGAD channel on the decay of quantum entanglement, Bell nonlocality, and quantum steering, especially one-way steering. The strength of channel correlation, the parameter of thermal photons, and the degree of squeezing on the dynamics of concurrence, Bell parameter, and critical radius are analyzed by numerical examples. Then we study the resilience of local WM and QMR to the aforementioned nonlocal quantum correlations in uncorrelated and completely correlated SGAD channels, respectively. Our results show that the decoherence on nonlocal quantum correlations can be weakened by adding correlation strength and squeezing degree or decreasing thermal photon parameter. And in both channels, nonlocal quantum correlations can be successfully recovered via WM and QMR. Moreover, in the completely correlated SGAD channel, the recovered correlation may be much stronger than their initial one. Different from entanglement and Bell nonlocality, the decay and revival of quantum steering are directional. The presented results provide useful references for applying nonlocal quantum correlations in noisy environments.

**Figure 1:**
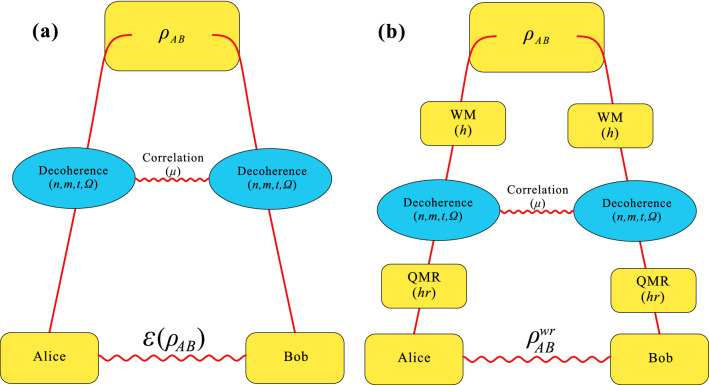
(Color online) (**a**) Evolution of nonlocal quantum correlations in noise channel. Two-qubit system state $\rho _{AB}$ are sent to Alice and Bob through a correlated SGAD channel $\varepsilon$. Decoherence in the quantum channel weakens the nonlocal quantum correlations of the output state between Alice and Bob $\varepsilon (\rho _{AB})$. (**b**) Recovering nonlocal quantum correlation from decoherence. The weakening of nonlocal quantum correlations caused by correlated SGAD channel can be recovered by sequentially performing local WM and QMR on the subsystem state before and after they undergo the channel.

## Result

### Channel model and state dynamics

The basic task of quantum information is to propagate quantum states from one observer to another through channels. In particular, we consider a scenario depicted in Fig. 1a where a two-qubit entangled state $\rho _{AB}$ is distributed to Alice and Bob through a correlated SGAD channel with the correlation strength $\mu \in [0,1]$. Usually, a quantum channel $\varepsilon$ is defined mathematically as a completely positive trace-preserving (CPTP) linear map on input state $\rho _{AB}$. And the output state $\varepsilon (\rho _{AB})$ can be expressed as

1 $$\begin{aligned} \varepsilon (\rho _{AB})=(1-\mu )\varepsilon _{U}(\rho _{AB})+\mu \varepsilon _{C}(\rho _{AB}), \end{aligned}$$

where $\varepsilon _{U}$ and $\varepsilon _{C}$ denote uncorrelated and completely correlated channel, respectively.

In the operator-sum representation, the uncorrelated SGAD channel maps the input state $\rho _{AB}$ to $\varepsilon _{U}(\rho _{AB})$, which can be expressed as

2 $$\begin{aligned} \varepsilon _{U}(\rho _{AB})=\sum \limits _{i,j=1}^{6}U_{A_i, B_j}\rho _{AB}U_{A_i, B_j}^{\dagger }. \end{aligned}$$

In this case, the kraus operator $U_{A_i, B_j}=u_{A_i}\otimes u_{B_j}$ is a product of two local kraus operators $u_{A_i}$ and $u_{B_j}$. The nonzero matrix elements $u_{A_i}^{xy}$ and $u_{B_j}^{xy}$ of $u_{A_i}$ and $u_{B_j}$ in the *x*-th row and the *y*-th column are^52,53^

3 $$\begin{aligned} \begin{aligned}{}&u_{A_1}^{11}=u_{B_1}^{11} =\sqrt{\frac{n}{2 n+1}-q g^{n+\frac{1}{2}}+\frac{(n+1) g^{2 n+1}}{2 n+1}},\\&u_{A_2}^{21}=u_{B_2}^{21}=\sqrt{\frac{(n+1)(1-g^{2 n+1})}{2 n+1}-r g^{n+\frac{1}{2}}},\\&u_{A_3}^{22}=u_{B_3}^{22}=\sqrt{\frac{n+1}{2 n+1}-q g^{n+\frac{1}{2}}+\frac{n g^{2 n+1}}{2 n+1}},\\&u_{A_4}^{11}=u_{A_4}^{22}=u_{B_4}^{11}=u_{B_4}^{22}=\sqrt{q g^{n+\frac{1}{2}}},\\&u_{A_5}^{12}=u_{A_5}^{21}=u_{B_5}^{12}=u_{B_5}^{21}=\sqrt{r g^{n+\frac{1}{2}}},\\&u_{A_6}^{12}=u_{B_6}^{12}=\sqrt{\frac{n (1-g^{2 n+1})}{2 n+1}-r g^{n+\frac{1}{2}}}, \end{aligned} \end{aligned}$$

where $g=e^{-\Omega t}$, $q=\cosh (m \Omega t )$, and $r=\sinh (m \Omega t )$. $\Omega$ is the dissipation rate, which is associated with the spontaneous emission at zero temperature^54^. In our work, we set $\Omega =1$. *n* is associated with the thermal photons, *m* is the squeezing degree which satisfies $m < n + 1/2$. Especially, SGAD channel reduces to GAD channel when $m=0$, and further to AD channel when $m=n=0$.

Similarly, after the completely correlated SGAD channel, the output state $\varepsilon _{C}(\rho _{AB})$ can be expressed as

4 $$\begin{aligned} \varepsilon _{C}(\rho _{AB})=\sum \limits _{k=1}^{7} {C_{k}}\rho _{AB} {C_{k}}^{\dagger }. \end{aligned}$$

The nonzero matrix elements $C_{k}^{xy}$ of the kraus operators $C_{k}$ in the *x*-th row and the *y*-th column are^52,53^

5 $$\begin{aligned} \begin{aligned}{}&C_{1}^{11}=\sqrt{g^{n+1}},\quad C_{1}^{44}=\sqrt{g^{n}},\quad C_{1}^{22}=C_{1}^{33}=1,\\&C_{2}^{14}= \sqrt{\frac{n+1}{2 n+1}(1-g^{2 n+1})- r g^{n+\frac{1}{2}}},\\&C_{3}^{41}=\sqrt{\frac{n}{2n+1}(1-g^{2 n+1})- r g^{n+\frac{1}{2}}},\\&C_{4}^{11}= \sqrt{\frac{n}{2 n+1}+\frac{n+1}{2 n+1}g^{2 n+1}-(q-1)g^{n+\frac{1}{2}}-g^{n+1}},\\&C_{5}^{44}=\sqrt{\frac{n+1}{2 n+1}+\frac{n}{2 n+1}g^{2 n+1}- (q-1)g^{n+\frac{1}{2}}-g^{n}},\\&C_{6}^{11}=C_{6}^{44}=\sqrt{(q-1) g^{n+\frac{1}{2}}},\\&C_{7}^{14}=C_{7}^{41}=i \sqrt{r g^{n+\frac{1}{2}}}. \end{aligned} \end{aligned}$$

To investigate the effect of correlated SGAD channel on the behavior of quantum entanglement, Bell nonlocality, and quantum steering, especially the steering direction, we consider a class of one-way steering state

6 $$\begin{aligned} \rho _{AB}(p,\theta )=p \vert \psi (\theta )\rangle \langle \psi (\theta )\vert +(1-p){\mathbb {I}}_{A}/2\otimes \rho _{B}^{\theta }, \end{aligned}$$

where $\vert \psi (\theta )\rangle =\cos (\theta )\vert 11\rangle +\sin (\theta )\vert 00\rangle$, $\rho _{B}^{\theta } =\textrm{Tr}_{A} [\vert \psi (\theta )\rangle \langle \psi (\theta )\vert ]$ denotes the reduced state of Bob. It has been demonstrated that for $\theta \in [0,\pi /4]$ and $\cos ^{2}(2\theta )\geqslant \dfrac{2p-1}{(2-p)p^{3}}$, Bob is not capable of steering Alice; however, Alice can steer Bob for $p>1/2$^55^. Note that nonlocal quantum correlations are very fragile and easily destroyed by noise channels. In the following two subsections, we chose a two-way steerable initial state from Eq. (6) with $p=0.85$ and $\theta =0.4$ as an example to discuss the behavior of the aforementioned quantum correlations.

Submitting Eq. (6) to Eq. (1), one can get the output state $\varepsilon (\rho _{AB})$ after the correlated SGAD quantum channel, whose density matrix is X-type (see more details in the section I of Supplementary Information). Here, we adopt the generalized concurrence *C*, Bell parameter *B* and steering radii $R_{AB}$ as well as $R_{BA}$ as the measure of entanglement, Bell nonlocality, the steerability from Alice to Bob and the steerability from Bob to Alice, respectively (see more details in the section of “Methods”).

### Decay effects

**Figure 2:**
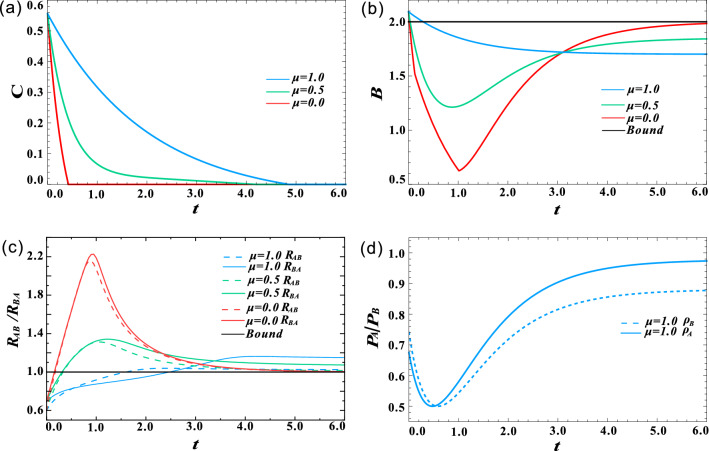
(Color online) Effect of channel correlation strength $\mu$ on the decay of nonlocal quantum correlations when $m=n=0$. (**a**) Concurrence *C* as a function of decoherence time *t* for different $\mu$. (**b**) Bell parameters *B* as a function of decoherence time *t* for different $\mu$. (**c**) Critical radii $R_{AB}$ and $R_{BA}$ as a function of decoherence time *t* for different $\mu$. (**d**) Purities $P_{A}$ and $P_{B}$ as a function of decoherence time *t* when $\mu =1$.

First, we investigated the effect of channel correlation strength $\mu$ on the performance of nonlocal quantum correlations. The thermal photon parameter *n* and squeezing degree *m* are set to zero, which means that the correlated SGAD channel acts as the correlated AD channel. The results for a two-way steerable state in the form of Eq. (6) with $p=0.85$ and $\theta =0.4$ are shown in Fig. 2. Here, quantum entanglement, Bell nonlocality and quantum steerability are quantified by concurrence *C*, Bell parameter *B* and steering radii $R_{AB}$ as well as $R_{BA}$, respectively. Their definitions are presented in the section of “Methods”. The evolution of *C*, *B* as well as $R_{AB}$ and $R_{BA}$ are plotted for three values of channel correlation strength: $\mu =0$ (red lines), $\mu =0.5$ (green lines) and $\mu =1.0$ (blue lines). Obviously, with the increase of $\mu$, the decay rate of concurrence *C*, Bell parameter *B*, and steering radii *R* slow down, indicating that correlated channel can protect nonlocal quantum correlations to a certain extent. For a fixed correlation strength $\mu$, steering radii $R_{AB}$ and $R_{BA}$ decay faster than concurrence *C*, and slower than Bell parameter *B*. The result again demonstrates that quantum steering is a correlation stronger than quantum entanglement, but weaker than bell nonlocality. Unlike *C*, which decays monotonically with increasing decoherence time *t*, *B*, $R_{AB}$ and $R_{BA}$ decrease first and then increase. The sudden change and revival phenomenon shown in Fig. 2b,c is due to AD noise decreasing the amplitude of excited state. As the AD channel strength increases, more qubits transition from excited state to ground state, and finally, the initial entangled mixed state decays into a separable pure ground state. Bell nonlocality and steerability can enhanced to some certain, but they cannot exceed the classical bound. And the increase of channel correlation strength $\mu$ can slow down the transition of qubits from excited state to ground state in the AD channel, thus affecting the sudden change and revival behavior. In addition, $R_{AB}$ increases faster than $R_{BA}$. When $\mu$ increases to 1, thought $R_{AB} > R_{BA}$ at $t=0$, it changes to $R_{AB} < R_{BA }$ once $t>0.5$. Especially, in the range of $t\in [1.6, 2.5]$, only Bob can steer Alice. It is worth noting that the one-way steerability from Bob to Alice is absent for the initial state $\rho _{AB}$ in the form of Eq. (6). To clarify this interesting phenomenon, we further respectively calculated the purity of Alice’s reduced state ($P_{A}=\textrm{Tr}[\rho _{A}^2]$) and that of Bob’s reduced state ($P_{B}=\textrm{Tr}[\rho _{B}^2]$)^56^, where $\rho _{A}= \textrm{Tr}_{B}[\varepsilon (\rho _{AB})]$ and $\rho _{B}= \textrm{Tr}_{A}[\varepsilon (\rho _{AB})]$. As shown in Fig. 2d, the decay trend of state purity is highly consistent with that of steerability. This is because, in the two-qubit system, the more entangled the composite state is, the more mixed the subsystem state is. Surprisingly, we found that as the decoherence time increases, the purity of the current reduced state can be increased to a larger value than that of the initial reduced state.

**Figure 3:**
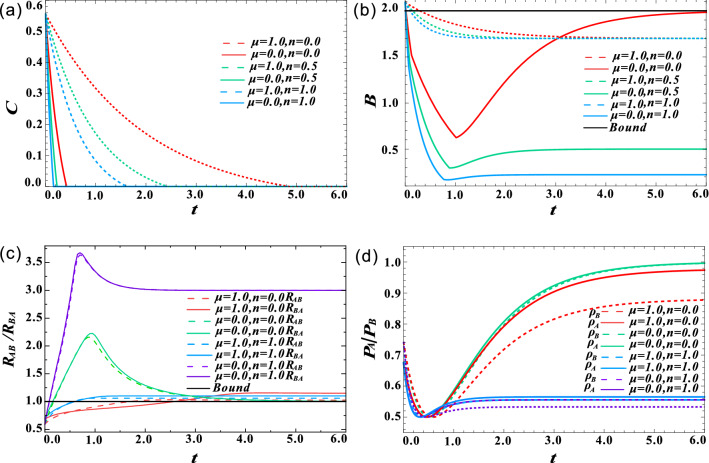
(Color online) Effect of thermal photon parameter *n* on the decay of nonlocal quantum correlations when $m=0$. (**a**) Concurrence *C* as a function of decoherence time *t* for different *n* and $\mu$. (**b**) Bell parameters *B* as a function of decoherence time *t* for different *n* and $\mu$. (**c**) Critical radii $R_{AB}$ and $R_{BA}$ a function of decoherence time *t* for different *n* and $\mu$. (**d**) Purities $P_{A}$ and $P_{B}$ as a function of decoherence time *t* when $\mu =1$ for different *n* and $\mu$.

**Figure 4:**
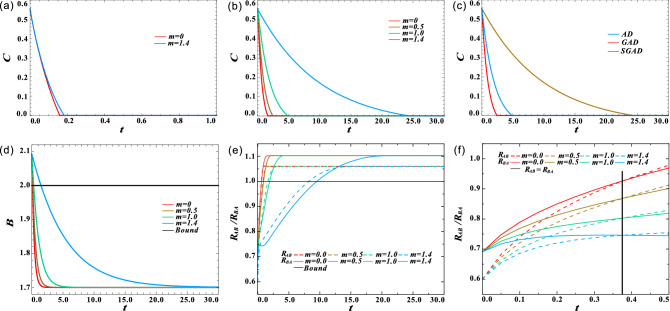
(Color online) Effect of squeezing degree *m* on the decay of nonlocal quantum correlations when $\mu =n=1$. (**a**) The effect of *m* on quantum entanglement when $\mu =0$. (**b**) Concurrence *C* as a function of decoherence time *t* for different *m*. (**c**) The evolution of Concurrence *C* under completely correlated AD ($\mu =1,m=n=0$), GAD ($\mu =1,m=0,n=1$) and SGAD ($\mu =1,m=1.4,n=1$) channels. (**d**) Bell parameters *B* as a function of decoherence time *t* for different *m*. (**e**) Critical radii $R_{AB}$ and $R_{BA}$ are a function of decoherence time *t* for different *m*. (**f**) The enlarged view of Fig. 4e when $t\in [0, 0.5]$, the black line $t=0.37$ denotes the time when $R_{AB}=R_{BA}$.

Then we show how thermal field photon number *n* affects the decay of quantum entanglement, Bell nonlocality, and quantum steering. Here, the squeezing parameter *m* is still equal to zero. The corresponding results are depicted in Fig. 3. Clearly, the thermal field photon number has a significant promotion effect on the decay of quantum nonlocal correlation. As shown in Fig. 3a–c, for both uncorrelated ($\mu =0$) and completely correlated ($\mu =1$) channel, quantum entanglement, Bell nonlocality, and quantum steering disappear faster with the increase of *n*. When $\mu =1$, as *n* increases from 0 to 1, the survival time of quantum entanglement decreases from $t=4.8$ to $t=1.6$, and when $\mu =0$, it decreases from $t=0.4$ to $t=0.08$, a little faster than former case. And the decay trend of quantum steering and Bell nonlocality is similar, except that the decay speed increases in turn. The evolutions of the reduced states’ purities for different *n* and $\mu$ are shown in Fig. 3d. It is also consistent with the decay trend of quantum steering shown in Fig. 3c. However, different to Fig. 2d, the purity at $t>0$ can not be larger than its original value when $n=1$.

Finally, we analyzed the effect of squeezing degree *m* on the evolution of nonlocal quantum correlations. To satisfy $m < n + 1/2$, we set $n=1$, the correlation survival time is limited to the case of $m = 1.5$. Here, we change *m* from 0 to 1.4. As shown in the Fig. 4a, when the correlation strength $\mu$ is small, the effect of *m* is not obvious, so we set $\mu =1$ in subsequent calculations. Figure 4b clearly shows that increasing *m* can prolong the survival time of quantum entanglement in noise channel. And the increased rate of survival time decreases, which is same as the effect of increasing $\mu$. When $m=1.4$, quantum entanglement can still survive when *t* reaches 25, which is about 15 times longer than the case of $m=0$. It clearly shows the advantage of the squeeze effect. As illustrated in Fig. 4c, the sudden death of entanglement in the completely correlated SGAD channel is later than that in the completely correlated AD and completely correlated GAD channels. This squeeze-induced protection can also be observed in Bell nonlocality and quantum steering, see Fig. 4d,e for more clarity. Different from the first two cases, even *m* takes a different value, $R_{AB}$ and $R_{BA}$ increase to be equal at the same decoherence time. Figure 4f is an enlarged view of Fig. 4e when $t\in [0, 0.5]$, the black line $t=0.37$ denotes the time when $R_{AB}=R_{BA}$, which does not depend on *m*.

### Revival effects

As mentioned above, using correlated or squeezed channels, nonlocal quantum correlations can be prevented from decoherence, but cannot be recovered. In this section, we will show how to recover the disappeared nonlocal quantum correlations via WM and QMR.

The scheme is depicted in Fig. 1b. Before the system qubits undergo the correlated SGAD channel, they are subject to WM locally, which partially collapses them towards a state that is less vulnerable to decoherence. The two-qubit local WM is an unsharp measurement, which can be expressed as

7 $$\begin{aligned} M_{w}=\begin{pmatrix} \sqrt{1-h}&{}0\\ 0&{}1 \end{pmatrix} \otimes \begin{pmatrix} \sqrt{1-h}&{}0\\ 0&{}1 \end{pmatrix}, \end{aligned}$$

where *h* is the strength of WM. After the correlated SGAD channel, local QMR is respectively operated on the subsystem. And the two-qubit local QMR operation can also be expressed as

8 $$\begin{aligned} M_{r}=\begin{pmatrix} 1&{}0\\ 0&{}\sqrt{1-hr} \end{pmatrix} \otimes \begin{pmatrix} 1&{}0\\ 0&{}\sqrt{1-hr} \end{pmatrix}, \end{aligned}$$

where *hr* is the strength of QMR. After WM, correlated SGAD channel and QMR, the final state $\rho _{wr}$ can be expressed as

9 $$\begin{aligned} \rho _{AB}^{wr}=M_{r}\varepsilon (M_{w}\rho _{AB}M_{w}^{\dagger })M_{r}^{\dagger }. \end{aligned}$$

Clearly, it is an X-type state, see more details in the section II of Supplementary Information. And the concurrence, Bell parameter, and critical radius can be obtained from Eqs. (10)–(12) by replacing $\varepsilon (\rho _{AB})$ with $\rho _{AB}^{wr}$.

**Figure 5:**
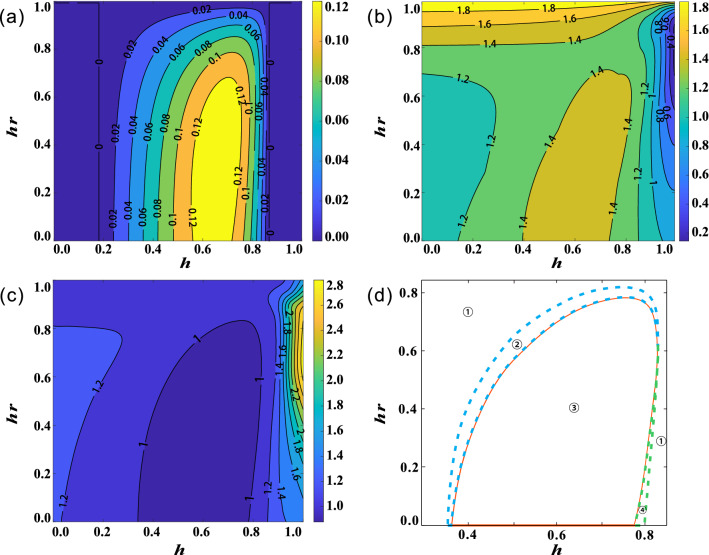
(Color online) Revival effects of nonlocal quantum correlations in uncorrelated SGAD channel ($\mu =0$). (**a**) The contour plot of concurrence *C*. (**b**) The contour plot of Bell parameter *B*. (**c**) The contour plot of critical radius $R_{AB}$. (**d**) The steering regions are parameterized by *h* and *hr*. *h* and *hr* represent the strength of WM and QMR, respectively.

First, we investigate the effect of WM and QMR on the revival of nonlocal quantum correlations in the presence of a completely uncorrelated SGAD channel, i.e., $u=0$. The rest channel parameters are set as $m=n=1$ and $t=0.2$. After passing through the channel, all the three types of nonlocal quantum correlations of the output state $\varepsilon (\rho _{AB})$ disappear. Figure 5a–c shows how *C*, *B* and $R_{AB}$ behave by employing WM and QMR. The relationship of $R_{AB}$, *h* and *hr* is presented in section II in Supplementary Information. The regions enclosed by $C=0$, $B=2$, $R_{AB}=1$ and $R_{BA}=1$ represent the existence of quantum entanglement, Bell nonlocality, Alice to Bob steering, and Bob to Alice steering, respectively.

Obviously, with some appropriate measurement strengths, the disappeared nonlocal quantum correlations can be recovered except for Bell nonlocality. For example, quantum entanglement reappears in the region of $h\in [0.17,0.87]$ and $hr\in [0,0.99]$, *C* reaches the maximum value 0.137 when $h=0.69$ and $hr=0.46$. Combining Fig. 5c and Fig. R1(a) in the section IIIof Supplementary Information, we can obtain different kinds of steering regions. As shown in Fig. 5d, the states located in regions labeled 1–4 are, in turn, no-way steering, only Alice can steer Bob, two-way steering and only Bob can steer Alice. By flexibly tuning *h* and *hr*, the steerability of $\rho _{AB}^{wr}$ can vary among no-way steering, one-way steering, and two-way steering.

**Figure 6:**
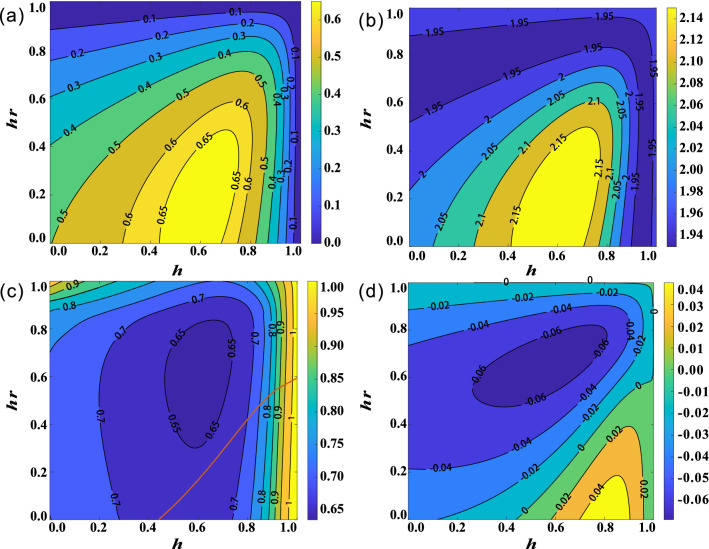
(Color online) Revival effects of nonlocal quantum correlations in completely correlated SGAD channel ($\mu =1$). (**a**) The contour plot of concurence *C*. (**b**) The contour plot of Bell parameter *B*. (**c**) The contour plot of critical radius $R_{AB}$. (**d**) The contour plot of relative purity $\Delta P=P_{A}-P_{B}$. *h* and *hr* represent the strength of WM and QMR, respectively.

To recover the disappeared Bell nonlocality, we further take the completely correlated SGAD channel into consideration. For comparison with the uncorrelated channel, the channel parameters *m*, *n*, and *t* are same as above. As shown in Fig. 6a–c, there are some regions that satisfy $C>0$, $B>0$, and $R_{AB}<1$, respectively, which indicates that WM and QMR can effectively recover the corresponding types of nonlocal quantum correlations. Interestingly, we found a super-recover phenomenon, that is, with the optimal WM and QMR, *C* can recover to the maximum value of 0.68, which is about 17% higher than the concurrence of the initial state $\rho _{AB}(p=0.85,\theta =0.4)$. In addition, the value of *B* can be 10 percent larger than the classical bound. Though two-way steering can be observed over the full ranges of *h* and *hr*, the relative steerability of Alice and Bob can be changed by tuning *h* or *hr*. As shown in Fig. 6c and Fig. R1(b) in the section III of Supplementary Information, when *h* and *hr* are on the left side of the red curve, the steerability of Bob is larger than that of Alice. However, when they are on the right side, the result is just the opposite. We also investigated the effect of WM and QMR on the relative purity of the reduced state $\Delta P=P_{A}-P_{B}$, the result is shown in Fig. 6d. Clearly, when *h* and *hr* are located on the left of the $\Delta P=0$, $P_{A}>P_{B}$, otherwise, $P_{A}<P_{B}$. Their results are consistent with the change of steerability.

## Conclusion

To summarize, we have investigated the decay of quantum entanglement, Bell nonlocality, and quantum steering under the correlated SGAD channel. These nonlocal quantum correlations are quantified by concurrence *C*, Bell parameter *B*, critical radii $R_{AB}$ and $R_{BA}$, respectively. We mainly analyze the roles of channel correlation strength $\mu$, the thermal photon parameter *n*, and the squeezing degree *m*. The results show the effects of correlation and squeezing reduce decoherence, however, the effect of thermal photon increases decoherence. And as the increase of $\mu$ and *m* or the decrease of *n*, the change of the decrease speed of *C* and *B* as well as the change of the increased speed of $R_{AB}$ and $R_{BA}$ become large. What’s more, $R_{AB}$ changes faster than $R_{BA}$, and a new one-way steering scenario from Bob to Alice appears.

Then we propose a scheme that utilizes local WM and QMR to revive nonlocal quantum correlations after decay occurs. By adjusting the strengths of WM and QMR, quantum entanglement, Bell nonlocality, and quantum steering can be recovered or even enhanced, especially in the completely correlated SGAD channel. This finding also opens up the possibility of developing optimal WM and QMR to perfectly distinguish uncorrelated transition from correlated transition.

It should be noted that whether in the process of decay or revival, the changes of *C*, $R_{BA}$, $R_{AB}$ and *B* slowed down in turn, which indicates that quantum steering is a state property that is more restrictive than quantum entanglement, and yet more general than Bell nonlocality. And the steering direction can be manipulated flexibly by adjusting the parameters of noise channel or the strengths of WM and QMR. The presented results provide useful references for understanding nonlocal quantum correlation properties of quantum state which interacts with noise environment, and also have potential applications in asymmetric quantum information processing exploiting quantum steering as a valuable resource.

## Methods

In this paper, quantum entanglement, Bell nonlocality, and quantum steering are quantified by concurrence, Bell parameter, and critical radius, respectively. It is easy to show that after the correlated SGAD quantum channel, the initial state shown in Eq. (6) evolves to an X-type state (see more details in the section I of Supplementary Information). The concurrence of the X-type output state $\varepsilon (\rho _{AB})$ can be expressed as^57^

10 $$\begin{aligned} C=2\max \lbrace 0,c_{1}, c_{2}\rbrace , \end{aligned}$$

where $c_{1}=|\varepsilon (\rho _{AB})_{14}|-\sqrt{\varepsilon (\rho _{AB})_{22}\varepsilon (\rho _{AB})_{33}}$, $c_{2}=|\varepsilon (\rho _{AB})_{23}|-\sqrt{\varepsilon (\rho _{AB})_{11} \varepsilon (\rho _{AB})_{44}}$, $\varepsilon (\rho _{AB})_{ij}$ is the matrix element of $\varepsilon (\rho _{AB})$. And $\varepsilon (\rho _{AB})$ is entangled if $C >0$.

And the Bell nonlocality can be tested by violating the Bell Clauser–Horne–Shimony–Holt (Bell-CHSH) inequality^58–60^. The Bell parameter of the X-type output state $\varepsilon (\rho _{AB})$ can be expressed as

11 $$\begin{aligned} B=2 \max \left\{ \sqrt{b_{1}+b_{2}}, \sqrt{b_{1}+b_{3}}\right\} , \end{aligned}$$

where $b_{1}=4(\left| \varepsilon (\rho _{AB})_{14}\right| +\left| \varepsilon (\rho _{AB})_{23}\right| )^{2}$, $b_{2}$ $=4(\left| \varepsilon (\rho _{AB})_{14}\right| -\left| \varepsilon (\rho _{AB})_{23}\right| )^{2}$, $b_{3}=(\varepsilon (\rho _{AB})_{11}-\varepsilon (\rho _{AB})_{22}-$ $\varepsilon (\rho _{AB})_{33}+\varepsilon (\rho _{AB})_{44})^{2}$. The state $\varepsilon (\rho _{AB})$ is Bell nonlocal if $B > 2$.

To capture the one-way steering, the steerability is quantified by the critical radius, which represents a necessary and sufficient steering criterion. The critical radius from Alice to Bob is defined as^61–63^

12 $$\begin{aligned} R_{AB}=\max _{\eta }\lbrace \eta >0:\rho ^{\eta }_{AB} \ \text {is unsteerable} \rbrace , \end{aligned}$$

where $\rho ^{\eta }=\eta \varepsilon (\rho _{AB})+(1-\eta ){\mathbb {I}}_{A}/2\otimes \rho _{B}$, $\rho _B= \textrm{Tr}_{A}[\varepsilon (\rho _{AB})]$ denotes the reduced state of Bob. Geometrically, $1- R_{AB}$ measures the distance from $\varepsilon (\rho _{AB})$ to the surface of unsteerable or steerable states relatively to $({\mathbb {I}}_{A} \otimes \rho _B)/2$^61–63^. The steering task from Alice to Bob is successful if $R_{AB}<1$. Otherwise, the task fails if $R_{A B}\ge 1$. Similarly, the steerability from Bob to Alice can be quantified by the critical radius $R_{BA}$.

## Supplementary Information

Supplementary Information.

## Data Availability

The datasets used and analysed during the current study available from the corresponding author on reasonable request.
