# Supplementary material for: Protecting nonlocal quantum correlations in correlated squeezed generalized amplitude damping channel
Source: Sci Rep. 2022 Nov 28;12:20481. doi: 10.1038/s41598-022-24789-z (PMC9705301; doi:10.1038/s41598-022-24789-z)
Supplement: Supplementary file 1 — Supplementary Information. [file 41598_2022_24789_MOESM1_ESM.pdf]

Supplementary information for Protecting nonlocal quantum correlations in correlated squeezed generalized amplitude damping channel

Shuo Wang[1], *Xing-Hong Han*[1], *Wei-Chen Li*[1], *Tian Qian*[1], *Xuan Fan*[1], *Ya Xiao*[1], and *Yong-Jian Gu*[1]

[1]*College of Physics and Optoelectronic Engineering, Ocean University of China, Qingdao* 266100, *People's Republic of China*

◇◇

I. OUTPUT STATE AFTER CORRELATED SGAD CHANNEL

When the initial state $\rho_{AB} = p|\psi(\theta)\rangle\langle\psi(\theta)| + (1-p)\mathbb{I}_A/2 \otimes \rho_B^\theta$ goes through the correlated SGAD, the nonzero matrix elements $\varepsilon(\rho_{AB})_{xy}$ of the output state $\varepsilon(\rho_{AB})$ in the x-th row and the y-th column are

$$
\begin{aligned}
&\varepsilon(\rho_{AB})_{11} = g^2[(1-\mu)\left((2n+1)(p+1)\cos(2\theta) + (2n+1)^2 p + 1\right) g^{4n} \\
&+ ((2n+1)(p+1)(2n+\mu)\cos(2\theta) + \mu(2n(p-1)+p+1) + 4n)g^{2n-1} \\
&+ 2n(2np\mu + 2n + p\mu + \mu)g^{-2}]/4(2n+1)^2; \\
&\varepsilon(\rho_{AB})_{22} = g^2[(1-\mu)(2g^{2n-1} + 4n(1+n)g^{-2} - (1+(1+2n)^2 p)g^{4n}) \\
&+ (2n+1)[(1-\mu)\cos(2\theta)g^{2n}\left(2(n(p-1)+p)g^{-1} - (p+1)g^{2n}\right) \\
&+ 2(2n+1)(p-1)\mu\sin^2(\theta)g^{-2}]]/4(2n+1)^2; \\
&\varepsilon(\rho_{AB})_{33} = 2(2n+1)^2(1-p)\mu\cos^2(\theta) + (1-\mu)g^2[4n(n+1)g^{-2} \\
&- (2(2n+1)(n(p-1)-1)\cos(2\theta) - 2)g^{2n-1} \\
&- \left((2n+1)(p+1)\cos(2\theta) + (2n+1)^2 p + 1\right) g^{4n}]/4(2n+1)^2; \\
&\varepsilon(\rho_{AB})_{44} = g^2[(1-\mu)\left((2n+1)^2 p + 1\right) g^{4n} \\
&- (\mu(2n(p-1)+p-3) + 4(n+1))g^{2n-1} + 2(n+1)(\mu(2np+p-1) + 2(n+1))g^{-2} \\
&- (2n+1)(p+1)((\mu-1)g^{2n} + (2n-\mu+2)g^{-1})\cos(2\theta)g^{2n}]/4(2n+1)^2; \\
&\varepsilon(\rho_{AB})_{14} = \varepsilon(\rho_{AB})_{41} = \frac{1}{2}p\sin(2\theta)[\mu g^{-m+n+\frac{1}{2}} + (1-\mu)\left(q^2+r^2\right) g^{2n+1}]; \\
&\varepsilon(\rho_{AB})_{23} = \varepsilon(\rho_{AB})_{32} = pqr(1-\mu)\sin(2\theta)g^{2n+1}.
\end{aligned} \tag{1}
$$

Here, $g = e^{-\Omega t}$, $q = \cosh(m\Omega t)$, $r = \sinh(m\Omega t)$.

Clearly, the non zero elements $\varepsilon(\rho_{AB})_{xy}$ are along the diagonal and anti-diagonal and satisfy the unit trace condition $\mathrm{Tr}\varepsilon(\rho_{AB})_{xx} = 1$. Therefore, $\varepsilon(\rho_{AB})$ is a X-type matrix like state.

II. FINAL STATE AFTER THE SEQUENTIAL WM, SGAD CHANNEL AND QMR

When the initial state $\rho_{AB} = p|\psi(\theta)\rangle\langle\psi(\theta)| + (1-p)\mathbb{1}_A/2 \otimes \rho_B^\theta$ sequentially undergoes WM, SGAD channel and QMR, the nonzero matrix elements $(\rho_{AB}^{wr})_{xy}$ of the final state ρ_{AB}^{wr} in the x-th row and the y-th column are

$$
\begin{aligned}
&(\rho_{AB}^{wr})_{11} = \mu(1+2n)(1+p)[n((h-2)h+2) + ((h-2)h(n+1)+1)g^{2n+1} \\
&+ \cos(2\theta)\left((h-2)hn + ((h-2)h(n+1)+2n+1)g^{2n+1}\right)] + (1-\mu)[2(h-1) \\
&* \left(n+(n+1)g^{2n+1}\right)\cos^2(\theta)\left(n(hp+h-2) + (p((h-2)n+h-1)+hn+h-1)g^{2n+1}\right) \\
&- 2n\left(g^{2n+1}-1\right)\sin^2(\theta)\left(n(h(p-1)+2) + (h(n+1)(p-1)-2np-p+1)g^{2n+1}\right)]/\Lambda; \\
&(\rho_{AB}^{wr})_{22} = (1-hr)[2\mu(h-1)(2n+1)^2(p-1)\sin^2(\theta) + (1-\mu)[n(n+1)(h(hp+h-4)+4) \\
&- \left(p((h-2)n+h-1)^2 + (hn+h-1)^2\right)g^{4n+2} \\
&+ \left(h^2(n+1)(p+1) - h(2n(p+1)+p+3)+2\right)g^{2n+1} \\
&+ \cos(2\theta)[(h-2)hn(n+1)(p+1) - (p+1)((h-2)n+h-1)(hn+h-1)g^{4n+2} \\
&+ \left(h^2(n+1)(p+1) - h(2n(2n(p-1)+3p-1)+3p+1) + 2(2n+1)(n(p-1)+p)\right)g^{2n+1}]]]/\Lambda; \\
&(\rho_{AB}^{wr})_{33} = (1-hr)[2\mu(h-1)(2n+1)^2(p-1)\cos^2(\theta) + (1-\mu)[2(1-h)\left(n+(n+1)g^{2n+1}\right) \\
&* \cos^2(\theta)\left((n+1)(2-hp-h) + (p((h-2)n+h-1)+hn+h-1)g^{2n+1}\right) \\
&+ 2n\left(g^{2n+1}-1\right)\sin^2(\theta)[(n+1)(h(1-p)-2) \\
&+ (h(n+1)(p-1)-2np-p+1)g^{2n+1}]]]/\Lambda; \\
&(\rho_{AB}^{wr})_{44} = (1-hr)^2[\mu(2n+1)(p+1)[((h-2)h+2)(n+1) \\
&- ((h-2)h(n+1)+1)g^{2n+1} + \cos(2\theta)\left((h-2)h(n+1) - ((h-2)h(n+1)+2n+1)g^{2n+1}\right)] \\
&+ (1-\mu)[2(1-h)(n+1)\left(1-g^{2n+1}\right)[(n+1)(h(-p)-h+2) \\
&+ (p((h-2)n+h-1)+hn+h-1)g^{2n+1}]\cos^2(\theta) - 2\left(n+1+ng^{2n+1}\right) \\
&* [(n+1)(h(1-p)-2) + (h(n+1)(p-1)-2np-p+1)g^{2n+1}]\sin^2(\theta)]]/\Lambda; \\
&(\rho_{AB}^{wr})_{14} = (\rho_{AB}^{wr})_{41} = 2(1-h)(1-hr)(2n+1)^2 p\sin(2\theta)\left(\mu g^{-m+n+\frac{1}{2}} + (1-\mu)\left(q^2+r^2\right)g^{2n+1}\right)/\Lambda; \\
&(\rho_{AB}^{wr})_{23} = (\rho_{AB}^{wr})_{32} = 4(1-h)(1-hr)(2n+1)^2 pqr(1-\mu)\sin(2\theta)g^{2n+1}/\Lambda.
\end{aligned}
\tag{2}
$$

Here

$$
\begin{aligned}
\Lambda &= 4((hr-2)n+hr-1)^2+hr^2(1-\mu)g^{4n+2}\left((2n+1)^2p+1\right) \\
&+2hr\mu(hr(n+1)(2np+p-1)+(2n+1)(1-p)) \\
&-hrg^{2n+1}(hr\mu(2n(p-1)+p-3)+4hr(n+1)-2(2n+1)((p-1)\mu+2)) \\
&+h^2(p+1)[2hr(n+1)(2n+1)\left(g^{2n+1}-1\right)+(2n+1)^2 \\
&+hr^2(n+1)\left(g^{2n+1}(-2n+\mu-2)-(n+1)(\mu-1)g^{4n+2}+n\mu+n+1\right)] \\
&+2h[hr(2n+1)\left(-\left(g^{2n+1}(2n(p+1)+(p-1)\mu+p+3)\right)+4n+(p-1)\mu+4\right)-2(2n+1)^2 \\
&+hr^2(1+n)[g^{2n+1}(2n(p+1)+p-2\mu+3)-2 \\
&+(\mu-1)g^{4n+2}(2np+p+1)-2(np\mu+n)-p\mu+\mu]] \\
&-(1+p)[hrg^{2n+1}((h-2)h(n+1)+2n+1)(hr(2n-\mu+2)-4n-2) \\
&+hr^2(\mu-1)g^{4n+2}((h-2)n+h-1)(hn+h-1) \\
&+(h-2)h\left(hr^2(-n)(n+1)\mu-((hr-2)n+hr-1)^2\right)]\cos(2\theta),
\end{aligned}
$$

$$g=e^{-\Omega t}, \quad q=\cosh(m\Omega t), \quad \text{and} \quad r=\sinh(m\Omega t).$$

Clearly, the non zero elements $(\rho_{AB}^{wr})_{xy}$ are along the diagonal and anti-diagonal and satisfy the unit trace condition $\mathrm{Tr}(\rho_{AB}^{wr})_{xx}=1$. Therefore, ρ_{AB}^{wr} is a X-type matrix like state.

III. REVIVAL EFFECT OF WM AND QMR ON THE STEERABILITY FROM BOB TO ALICE

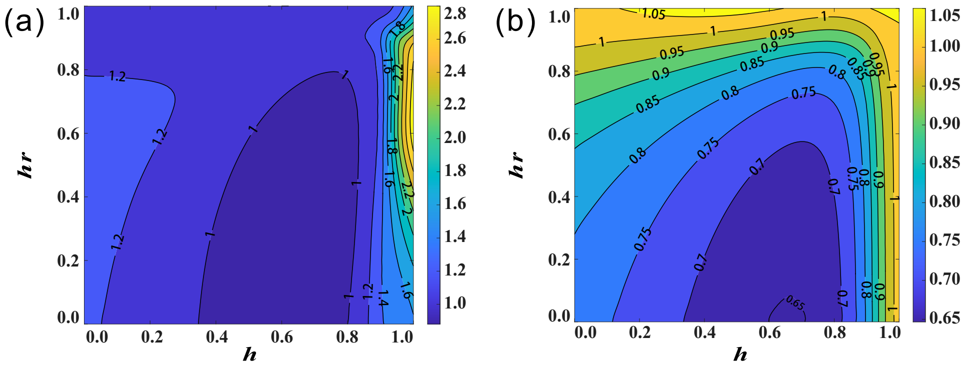

FIG. R1. (color online) The contour plot of critical radius R_{BA} in uncorrelated SGAD channel (a) and correlated SGAD channel (b). h and hr represent the strength of WM and QMR, respectively.
